# Diet Containing Soy Protein Concentrate With Low and High Isoflavones for 9 Weeks Protects Against Non-alcoholic Fatty Liver Steatosis Using Obese Zucker Rats

**DOI:** 10.3389/fnut.2022.913571

**Published:** 2022-06-22

**Authors:** Reza Hakkak, Beverly Spray, Elisabet Børsheim, Soheila Korourian

**Affiliations:** ^1^Department of Dietetics and Nutrition, University of Arkansas for Medical Sciences, Little Rock, AR, United States; ^2^Department of Pediatrics, University of Arkansas for Medical Sciences, Little Rock, AR, United States; ^3^Arkansas Children's Research Institute, Little Rock, AR, United States; ^4^Arkansas Children's Nutrition Center, Little Rock, AR, United States; ^5^Department of Pathology, University of Arkansas for Medical Sciences, Little Rock, AR, United States

**Keywords:** obesity, non-alcoholic fatty liver disease, high and low soy protein concentrate, isoflavones, Zucker rats

## Abstract

Non-alcoholic fatty liver disease (NAFLD), is one of the main liver diseases in the US and the world which often is related to obesity. Previously, we reported short- and long-term consumption of soy protein isolate diet with high isoflavones can reduce liver steatosis in the male and female obese Zucker rat model. However, the effects of high vs. low soy isoflavones on NAFLD is less known. The objectives of the present study were to examine the role of isoflavones levels in soy protein concentrate diets on protection against NAFLD in an obese rat model. Forty-two 6-week old lean (L, *n* = 21) and obese (O, *n* = 21) Zucker rats were randomly assigned to 1 of 3 dietary groups: casein diet (C = control), soy protein concentrate with low isoflavones (LIF), or soy protein concentrate with high isoflavones (HIF) for 9 weeks. Rats were weighed twice weekly. After 9 weeks, rats were sacrificed and samples of livers were taken for histopathological analysis. Serums were collected to measure ALT and AST levels. Results indicate that obese rats gained significantly more weight than lean rats for all three diet groups (*P* < 0.001). No significant difference in body weight between LC, LLIF and LHIF was noted. However, the OLIF and OHIF rats gained significantly more weight than OC rats (*P* < 0.001). Liver steatosis scores were significantly greater in obese rats compared to lean rats (*P* < 0.001). The OLIF and OHIF-fed rats had significantly reduced steatosis scores than OC rats (*P* = 0.013 and *P* < 0.001, respectively). The serum ALT levels were significantly greater in OC, OLIF and OHIF compared to LC, LLIF and LHIF, respectively (*P* < 0.001, *P* < 0.001, and *P* = 0.011). AST serum levels were greater in OC and OLIF compared to LC and LLIF, respectively (*P* = 0.001 and *P* = 0.022). In summary, we found that soy protein concentrate with isoflavones protects against liver steatosis and the protection is greater with a higher concentration of isoflavones.

## Introduction

The rate of obesity is growing in the US and worldwide. According to the World Health Organization (WHO), the world number of obese people has tripled between 1975 and present in the world^1^. In 2016, ~1.9 billion adults were overweight, including over 650 million who were obese^1^. The latest report from the Center for Disease Control (CDC) indicates that the US obesity rate jumped from 30.5 to 42.4% between 1997/1998 and 2017/2018, and the rate of severe obesity increased from 4.7 to 9.2% during the same time (1)^2^. Childhood obesity is a serious problem in the United States, putting children and adolescents at risk for poor health. Between 2017 and 2018, the prevalence of obesity was 19.3% and affected about 14.4 million children and adolescents (1)^2^. There is a link between obesity and risk of development of several chronic diseases such as heart disease, stroke, type 2 diabetes, liver diseases such as non-alcoholic fatty liver disease (NAFLD), and certain types of cancer (2, 3).

The NAFLD is one of the main liver diseases in the US and world, and is often related with obesity and diabetes. Approximately 1 in 10 American children have NAFLD; it is found in 33% to 58% of children who are obese (4). The NAFLD mortality in is significantly higher than in the healthy people, with complications from NAFLD is normal cause of death. Obesity can accumulate fat in the abdominal area which can affects both fat and glucose metabolism. Human with NAFLD are at higher risk of developing insulin resistant diabetes. NAFLD is the leading cause of liver disease both in adolescents and adults in the US, and NAFLD risk has increased as the rates of obesity have risen over the last three decades. With obesity on the rise worldwide, NAFLD is one of the most common liver diseases in adults (5–9).

Soy foods and soybean constituents have been widely investigated for their preventive role in chronic disease, which is attributed to their major physiological functions, such as cholesterol lowering, anti-obesity, antihypertensive, immunity regulation, lipid lowering, anti-carcinogenic, anticoagulant, anti-osteoporosis, and antioxidant functions (10). Several studies have reported that consumption of soy protein can decrease serum cholesterol and triglycerides and also reduce accumulation of cholesterol and triglycerides in the liver which can lead to reduction of liver steatosis (11–13). Soy protein has bioactive compounds such as isoflavones which have the capability to lower lipids in liver (14, 15). Soy protein isolate has more than 90% protein with high isoflavones but soy protein concentrate contains 70% protein and has lower isofavones than soy protein isolate. A feeding study using soy protein and obese Zucker diabetic rats (an animal model for type II diabetes) showed lowered serum insulin and small increases in hepatic sterol regulatory element binding protein-1 (SREBP-1) mRNA; long-term soy protein feeding prevented hyperinsulinemia and decreased SREBP-1 mRNA, which led to lower hepatic triglycerides and cholesterol in obese Zucker diabetic rats (16). One study reported that including soy isoflavones to a casein-based diet reducedliver triglycerides compared to the casein diet as control. They showed that the lowered plasma aspartate transaminase (AST) and alanine transaminase (ALT) levels which had decreased plasmaalkaline phosphatase, bile acids, lowered plasma and liver mRNA levels of TNF-α, lower interleukin-1 β (IL-1 β) and monocyte chemoattractant protein-1, and an highered plasma anti-inflammatory fatty acid index (8). Previously, we used lean and obese zucker rats and fed either casein or soy protein isolate and reported that soy protein diet was associated with increased expression of Fatty Acid Synthase (FASN), Malic Enzyme 1 (ME1), 6PGD, Sterol Regulatory Element Binding Protein-1c (SREBP-1c) and SREBP-2 genes in the livers of obese but not lean rats. We concluded that soy protein consumption can counter hepatic steatosis while coincidently promoting hepatic lipogenic gene expression (17).

Previously, we reported short- and long-term consumption of soy protein isolate diet with high isoflavones can reduce liver steatosis in the male and female obese Zucker rat model (17–20). Several studies from our laboratory and others have shown by using male and female obese Zucker rats soy protein isolate diet had an increased body weight gain compared to control-fed obese rats on a casein-based diet (17–24). However, the effects of soy protein concentrate with low and high isoflavones levels on liver steatosis is less known. Therefore, the objectives of this study were to determine the effects of diet containing soy protein concentrate with either low or high isoflavones on liver steatosis and serum ALT or AST levels.

## Materials and Methods

### Ethics Statement

All animal care and procedures were approved by the University of Arkansas for Medical Sciences/Arkansas Children's Research Institute Institutional Animal Care and Use Committee and adhered to the institutional policies and procedures (Protocol code no. 3968; approved on December 20, 2019). The guidelines of the United States Department of Agriculture (USDA, Washington, DC, USA) Animal Welfare Act were followed to ensure that the care and use of animals were appropriate.

### Experimental Design

Forty-two (42), 6-weeks-old male lean or obese Zucker (*fa/fa*) rats were purchased from Charles River (Wilmington, MA). Diets were prepared by Envigo (Madison, WI) and soy protein concentrate (SPC) with low and high isoflavones were donated by The Archer-Daniels-Midland Company; Chicago, IL (ADM). The diet compositions are listed in Table 1. We used the American Institute of Nutrition Rodent Diet for growth (AIN-93 G diet) which is a purified diet with casein as main source of protein and soybean oil as source of fat We replaced soybean oil with corn oil. The protein source was either casein as control or soy protein concentrate with high isofavones or soy protein with low isoflavones. We used lean rats to compare the effects of obesity (lean vs. obese) with 3 different diets on liver steatosis. Rats had *ad libitum* access to water and semi-purified diet similar to AIN-93G diet with dietary protein: (1) casein for the control diet, (2) soy protein diet with low isoflavone content of ~0.154 mg total isoflavones/g protein (Arcon SJ; ADM; Decatur, IL), or (3) soy protein diet with high isoflavone content of ~2.153 mg total isoflavones/g protein (Arcon SM; ADM; Decatur, IL). The low isoflavone soy protein diet has a negligible total 0.154 mg isoflavones/g protein with a total aglycone component of ~0.16 (genistein, 0.15 mg/g protein; daidzein, 0.011 mg/g protein; glycitin, LOD (below level of detection). The high isoflavone soy protein has a total isoflavone content of 2.153 mg/g protein with a total aglycone component of ~1.72 mg/g protein (genistein, 0.382 mg/g protein; daidzein, 0.216 mg/g protein; glycitin, 0.005 mg/g protein). The casein-based diet is devoid of isoflavones; all diets will be isocaloric and isonitrogenous.

**Table 1:** Diet compositions.

**Ingredients (g/kg)**	**Casein (g/kg)**	**SPC low isoflavone (g/kg)**	**SPC high isoflavone**
Casein	200	–	–
Soy protein concentrate	–	268	268
L-cystine	3	1.2	1.2
L-methionine	–	2.2	2.2
Corn starch	397.5	379.9	379.9
Maltodextrin	132	132	132
Sucrose	100	100	100
Corn oil	70	70	70
Cellulose	50	–	–
AIN-93 G Min. mix	35	35	35
AIN-93 G Vit. mix	10	10	10
Choline bitartrate	2.5	2.5	2.5

*SPC, Soy protein concentrate*.

After 1 week of acclimation on AIN 93G diet, each group of rats (lean; *n* = 21 and obese; *n* = 21) were randomly assigned to 1 of 3 dietary groups (Table 1): casein as control (C; *n* = 7), soy protein concentrate (SPC) with low isoflavones (LIF, *n* = 7, <0.4 mg/g protein), and soy protein concentrate (SPC) with high isoflavones (HIF, *n* = 7 1.5–2.0 mg/g protein). Thus, we generated six groups of diets: lean control (LC), lean low isoflavone (LLIF), lean high isoflavone (LHIF), obese control (OC), obese low isoflavone (OLIF), and obese high isoflavones (OHIF). Rats were housed two per cage until they weighted 450 g, then were placed one per cage. Rats were in light–dark cycles of 12 h each, and had *ad libitum* access to feed and water. Rats were anesthetized with carbon dioxide and euthanized by decapitation after 9 weeks on the assigned diet. Livers were surgically removed and weighed, and samples taken for histopathological analysis. In addition, serum samples were collected for post biomarkers analysis. Levels of serum AST and ALT were measured to detect liver damage.

### Liver Histology and Weights

At the end of the experiment (9 weeks), livers were removed, and their weights were recorded. Liver weight was divided by final body weight to express liver weight as a percentage of body weight. Two 3-mm sections of each liver lobe were fixed in tissue cassettes and stored in 10% buffered formalin until histological examination. Upon examination, liver sections were cut, and stained with hematoxylin and eosin, and then they were examined by a pathologist in a blinded protocol. All of the liver sections were evaluated individually for the presence of microvesicular and macrovesicular steatosis. The steatosis was semiquantitated as a score of 0 to 4 in each case based on relative degree of steatosis within hepatocytes: (0) no steatosis; (1) <25%; (2) 25–50%; (3) >50–75% and (4) >75% as previously reported (25).

### Serum ALT and AST

Serum ALT and AST were measured using a COBAS INTEGRA® 400 Plus chemistry analyzer (Roche Diagnostics Corporation, Indianapolis, IN), and following the manufacturer's instructions.

### Statistical Analysis

Data for each outcome variable was assessed for normality using box-and-whisker plots and the Shapiro–Wilk test. The assumption of equal variance was assessed with Levene's test. Data are presented as mean ± standard deviation (SD). To determine if outcome variables differed between lean and obese rats and with or without low and high levels of isoflavones, a general linear model procedure was employed with treatment as the main effect. If a significant main effect of treatment was found, the statistical differences among the treatments were analyzed using contrast statements in the SAS GLM procedure. *P*-values from multiple contrasts were adjusted using Holm's procedure. Statistical tests were considered statistically significant at the 0.05 level. Analyses were performed in SAS software, version 9.4 (SAS Institute, Cary NC, USA).

## Results

**Figure 1:**
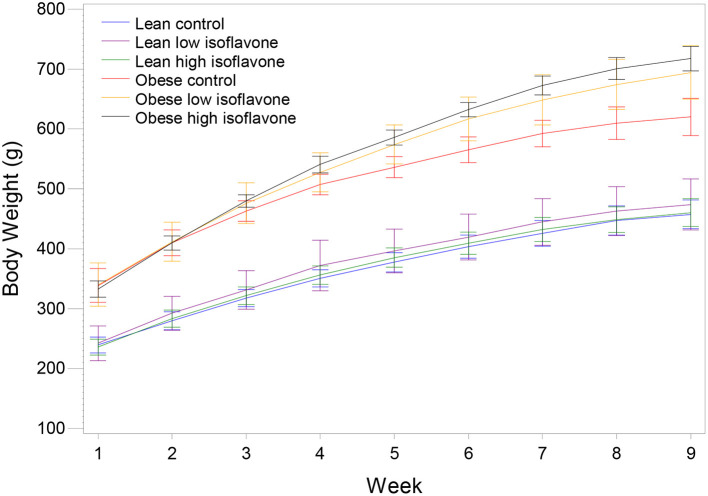
Mean body weight and standard deviation of lean and obese rats on the control and soy protein concentrate diets with low and high isoflavones content. *N* = 7 rats/group.

Rats from the three obese groups had similar initial weights, as well the rats from the three lean groups. During the experiment all rats gained weight in their respective dietary groups (Figure 1). The final mean body weights (BW), mean liver weights, liver weights as percentage of BW and steatosis scores are presented in Table 2. Table 3 contains the *p*-values for contrasts between obese and lean rats on low and high isoflavones diets. The mean BW of LLIF and LHIF-fed were not significantly different compared to lean-fed control diet. In contrast, obese rats fed SPC with high isoflavones (OHIF) gained more weight than obese control (OC) and obese rats fed SPC with low isoflavones (OLIF) after 9 weeks of diet (*P* < 0.001). There were no significant differences between body weights of OLIF vs OHIF group. Mean liver weights, liver weights as a percentage of BW, and steatosis scores are presented in Table 2. Liver weight was significantly greater in OC rats compared to obese rats on LIF and HIF diets (*P* < 0.001). Liver weights for obese rats on both LIF and HIF diets were significantly heavier than lean rats on LIF and HIF, respectively (*P* < 0.001). Liver weight as a percentage of BW was significantly greater in OC rats compared to obese rats on LIF and HIF (*P* < 0.001). While obese rats on LIF had significantly greater liver percentages then lean LIF rats, the lean and obese rats on HIF diets did not differ significantly. The steatosis scores in obese rats decreased with the increment of isoflavones levels in the diets (OC>OLIF>OHIF) at the end of 9 weeks of SPC feeding.

**Table 2:** Mean body weight (g) ± standard deviation, liver weight (g), liver weight as percent of body weight and steatosis score for lean and obese rats on soy protein concentrate and casein control diet.

	**Final body weight**	**Liver weight**	**Liver percent^a^**	**Steatosis score**
LC	457.3 ± 24.0	16.6 ± 1.4	3.6 ± 0.3	0.3 ± 0.5
LLIF	473.7 ± 42.5	16.3 ± 1.8	3.5 ± 0.6	0.1 ± 0.4
LHIF	460.3 ± 23.3	17.0 ± 2.8	3.7 ± 0.5	0.0 ± 0.0
OC	620.0 ± 31.1	45.9 ± 6.4	7.4 ± 1.1	3.0 ± 0.2
OLIF	694.4 ± 44.5	31.7 ± 6.8	4.6 ± 0.9	2.3 ± 0.8
OHIF	717.4 ± 20.2	29.0 ± 4.3	4.1 ± 0.7	1.6 ± 0.8

a *Liver weight as a percentage of final body weight. LC, lean control; LLIF, lean low isoflavones; LHIF, lean high isoflavones; OC, obese control; OLIF, obese low isoflavones; OHIF, obese high isoflavones*.

**Table 3:** Significance level (*p*-value) of pairwise contrasts between lean and obese rats on low and high diet isoflavones for final body weight, liver weight, liver weight as percent of body weight and steatosis score.

	**Final body weight**	**Liver weight**	**Liver percent^a^**	**Steatosis score**
LC vs. LLIF	0.3486	0.9051	0.5131	0.6047
LC vs. LHIF	0.8633	0.8581	0.9238	0.3032
LLIF vs. LHIF	0.4426	0.7658	0.4540	0.6047
OC vs. OLIF	**0.0001**	**<0.0001**	**<0.0001**	**0.0131**
OC vs. OHIF	**<0.0001**	**<0.0001**	**<0.0001**	**<0.0001**
OLIF vs. OHIF	0.1920	0.2615	0.1874	**0.0131**
LC vs. OC	**<0.0001**	**<0.0001**	**<0.0001**	**<0.0001**
LLIF vs. OLIF	**<0.0001**	**<0.0001**	**0.0027**	**<0.0001**
LHIF vs. OHIF	**<0.0001**	**<0.0001**	0.2707	**<0.0001**

a *Liver weight as a percentage of final body weight. LC, lean control; LLIF, lean low isoflavones; LHIF, lean high isoflavones; OC, obese control; OLIF, obese low isoflavones; OHIF, obese high isoflavones*.

*Bold means the p value were significant*.

**Figure 2:**
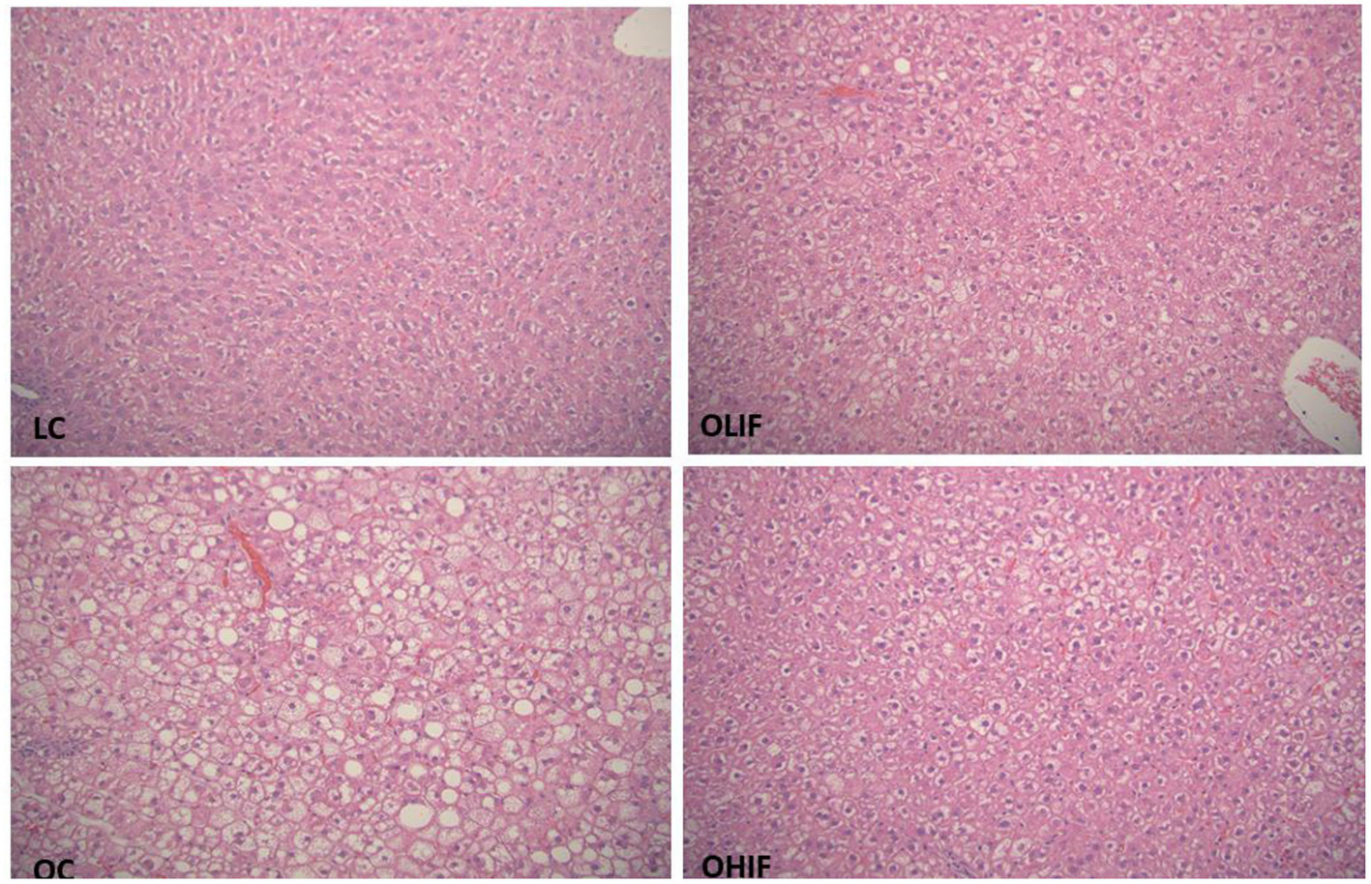
Liver Steatosis in LC, lean control; OC, obese control; OLIF, obese low isoflavones; OHIF, obese high isoflavones fed rats. LC image shows morphologic features of one of lean sample, there is no evidence of steatosis in any lean samples. OC image shows marked micro and macrovasicular steatosis present essentially in all three zones. OLIF image shows predominantly microvasicular steatosis, predominantly around portal zone. OHIF image shows predominantly microvasicular steatosis, predominantly around portal zone. All images are original at 20 x magnifying power.

The representative photomicrographs of hepatic parenchyma for LC, OC, OLIF and OHIF are shown in Figure 2. There were no significant difference with lean groups fed 3 different diets. We showed only LC steatosis in Figure 2 due there were no changes and had similar liver steatosis within lean groups. The steatosis score was significantly (*P* < 0.001) lower in obese rats fed with HIF compared to obese rats fed with LIF.

Mean serum levels of AST and ALT are presented in Table 4 and *p*-values of pairwise contrasts between lean and obese rats on low and high isoflavones diets are shown in Table 5. For both AST and ALT, OC rats had significantly greater serum levels compared to LC rats (*P* = 0.001 and *P* < 0.001). Obese rats on both LIF and HIF diets had significantly greater levels of ALT compared to lean rats on LIF and HIF, respectively (*P* < 0.001 and *P* = 011). For AST, only obese rats on a LIF diet had higher levels compared to the lean rats on LIF (*P* = 0.022). In lean rats, neither low nor high isoflavones diets yielded significant differences in AST or ALT serum levels.

**Table 4:** Mean ± standard deviation of aspartate aminotransferase (AST) and alanine transaminase (ALT) between lean and obese rats on low and high diet isoflavones.

	**Aspartate aminotransferase**	**Alanine transaminase**
LC	132.6 ± 51.3	35.0 ± 13.5
LLIF	156.9 ± 185.4	24.7 ± 19.3
LHIF	155.5 ± 36.8	34.0 ± 5.6
OC	325.0 ± 139.6	84.9 ± 35.2
OLIF	199.4 ± 36.6	58.0 ± 19.5
OHIF	213.7 ± 83.4	69.7 ± 28.1

*LC, lean control; LLIF, lean low isoflavones; LHIF, lean high isoflavones; OC, obese control; OLIF, obese low isoflavones; OHIF, obese high isoflavones*.

**Table 5:** Significance level (*p*-value) of pairwise contrasts between lean and obese rats on low and high diet isoflavones for aspartate aminotransferase and alanine transaminase.

	**Aspartate aminotransferase**	**Alanine transaminase**
LC vs. LLIF	0.6282	0.1105
LC vs. LHIF	0.3731	0.7230
LLIF vs. LHIF	0.1730	0.0538
OC vs. OLIF	0.1009	0.1594
OC vs. OHIF	0.1238	0.4613
OLIF vs. OHIF	0.9146	0.4933
LC vs. OC	**0.0010**	**0.0005**
LLIF vs. OLIF	**0.0219**	**0.0003**
LHIF vs. OHIF	0.2729	**0.0107**

*LC, lean control; LLIF, lean low isoflavones; LHIF, lean high isoflavones; OC, obese control; OLIF, obese low isoflavones; OHIF, obese high isoflavones*.

*Bold means the p value were significant*.

## Discussion

The histological of NAFLD changes from simple steatosis to steatosis with inflammation and cellular injury (steatohepatitis), fibrosis, and cirrhosis. NAFLD is of the main cause of liver diseases with a prevalence of up to 34% in the US (26, 27). The NFLAD prevalence increases to >50% in the obese population. NAFLD is associated with obesity, hyperlipidemia, and type 2 diabetes. The NAFLD patients are at risk of hepatocellular carcinoma (27). A study reported that the NAFLD patientshad a higher dead rate as compare to the general population, and liver disease was a main cause of mortality in NAFLD patients (28).

Fatty Zucker rat (*fa/fa*) is a well-known animal model for the study of genetic obesity and the associated fatty liver disease, becoming obese by 3 weeks of age (29, 30). Previously, we used obese Zucker rats and reported that soy protein isolate (SPI), which contains high levels of isoflavones (~90%), can exert protective properties for the liver against liver steatosis in obese Zucker rats (17–20, 31). Soy protein concentrate (SPC) contains lower levels of isoflavones (~70%) than SPI. However, the effects of low vs. high isoflavones SPC feeding on liver steatosis in an obese rat model are less known.

In the current study, we found (1) obese rats gained significantly more weight than lean rats for all three diet groups, (2) obese rats had significantly higher liver steatosis scores compared to lean rats, (3) obese soy protein-fed rats with high isoflavones (OHIF) and low isoflavones (OLIF) showed significantly lower levels of steatosis. In addition they showed significantly less macrovesicular steatosis as compared to the obese control fed-rats group (OC), with higher protection observed in obese soy-fed with high isoflavones compared to low isoflavones fed rats, and (4) serum ALT and AST levels were significantly greater in OC, OLIF and OHIF compared to LC, LLIF and LHIF, respectively and they were not different within either the 3 obese or the 3 lean groups.

Soy protein isolate has more than 90% protein with high isoflavones while soy protein concentrate contains 70% protein and has high and low isoflavones. In current study, we used soy protein concentrate with high and low isoflavones to investigate the effects of isoflavones within soy protein on non-alcoholic fatty liver in obese zucker rats. We reported that feeding SPI diet for 16 weeks with high isoflavones using the male and female obese Zucker rats decreased liver steatosis score, absolute liver weights, lower liver weight as percentage of body weight, and caused a higher body weight gain compared to obese rats fed a casein diet (17–20). Previously, we used similar study and reported that the energy intake of the obese soy fed-rats compared to obese casein-fed rats were only different at weeks 2 and 3 and no differences at end of experiment (19).

In agreement with our results, several studies including ours have reported that consumption of SPI can lower liver steatosis score (11–13). It is important to know which bioactive molecules in soy protein (genistein, daidzein and glycitein) are responsible for decreasing the lipid lowering of soy protein. Some reports indicated that soy protein with isoflavones reduced liver lipids, which can lowered cholesterol, and adding unconjugated soy isoflavones (genistein and daidzein) to a casein (CAS) diet decreased hepatic concentration of triglycerides compared to the casein diet alone (16, 32). Several studies have shown that isoflavones in soy protein are responsible, at least in part, for the reduction of liver steatosis (16, 32–35). A study by Liu et al. (36) using Sprague-Dawley rats fed high fat diet with oral isoflavones at 10 or 20 mg/kg reported that soy isoflavones are reducing liver steatosis and delaying the progression of NAFLD by inhibiting lipogenesis and promoting liver fatty acid oxidation (36). Another soy isoflavones component is daidzein which is unique compared to genistein and glycitein. Daidzein is metabolized by colonic bacteria into equol and O-desmethylangolensin (O-DMA); equol has a higher estrogenic effect compared to daidzein and O-DMA (37). However, it is estimated that only 25–60% of the human population have the gut bacteria profile capable of producing equol, with people from Asian populations trending toward the higher end of the range (37). We investigated whether or not the level of daidzein in soy protein isolate may be responsible for protection of liver steatosis. We used casein-based diet and fed obese rats either a high daidzein [match high isoflavones soy protein isolate (0.121 g/kg feed)] or low daidzein [match low isoflavones soy protein isolate (0.01 g/kg feed)] for 8 weeks. Comparing the low vs. high daidzein diet groups, we found no significant differences in mean for steatosis scores, liver weight, body weights, energy intake, or serum leptin levels. We concluded that daidzein, the main SPI isoflavonesmay not be responsible for reducing liver steatosis or increasing body weight in obese Zucker rats (38). These findings were in agreement with of Kim et al. (39) reporting that diet containing daidzein (0.1 g per kg of diet) did not have a significant effect on the total liver fatusing a high-fat (36% of total energy) diet-induced obese mice compared to mice on a high-fat diet without daidzein (39). Human studies reported that SPI improve insulin sensitivity (a known risk factor for liver steatosis) in postmenopausal women with abdominal obesity and metabolic syndrome (40). Recently, Xiong and Zhu reported that soy diet could significantly reduce HOMA IR and increase insulin in patients with non-alcoholic fatty liver disease (41). While there is no approved pharmacologic agent for the treatment of non-alcoholic fatty liver disease, there is a potential for liver steatosis protection by soy protein concentrate and soy protein isolate.

## Conclusion

We found that obesity increased liver steatosis and feeding nine (9) weeks of soy protein concentrate with isoflavones protects against liver steatosis. This protection is greater with a higher concentration of isoflavones in soy protein concentrate using an obese Zucker rat as a NAFLD model. Our data can provide additional benefit which shows soy protein concentrate consumption may reduce non-alcoholic fatty liver diseases in human.

## Data Availability Statement

The raw data supporting the conclusions of this article will be made available by the authors, without undue reservation.

## Ethics Statement

All animal care and procedures were approved by the University of Arkansas for Medical Sciences/Arkansas Children's Research Institute Institutional Animal Care and Use Committee and adhered to the institutional policies and procedures (Protocol code no. 3968; approved on December 20, 2019).

## Author Contributions

RH was involved in experimental design. RH, BS, EB, and SK were involved in data analysis and interpretation and writing and editing the manuscript. SK was involved in liver steatosis analysis. RH and BS were involved in writing the original draft preparation. All authors have read and agreed to the published version of this manuscript.

## Funding

This project was supported by USDA Grant Number 034036 to RH.

## Conflict of Interest

The authors declare that the research was conducted in the absence of any commercial or financial relationships that could be construed as a potential conflict of interest.

## Publisher's Note

All claims expressed in this article are solely those of the authors and do not necessarily represent those of their affiliated organizations, or those of the publisher, the editors and the reviewers. Any product that may be evaluated in this article, or claim that may be made by its manufacturer, is not guaranteed or endorsed by the publisher.

## Footnotes

^1^https://www.who.int/news-room/fact-sheets/detail/obesity-and-overweight (accessed March 19, 2022).

^2^https://www.cdc.gov/obesity/data/adult.html (accessed March 19, 2022).
